# Melatonin Suppresses Microglial Necroptosis by Regulating Deubiquitinating Enzyme A20 After Intracerebral Hemorrhage

**DOI:** 10.3389/fimmu.2019.01360

**Published:** 2019-06-14

**Authors:** Jianan Lu, Zeyu Sun, Yuanjian Fang, Jingwei Zheng, Shenbin Xu, Weilin Xu, Ligen Shi, Shuhao Mei, Haijian Wu, Feng Liang, Jianmin Zhang

**Affiliations:** ^1^Department of Neurosurgery, The Second Affiliated Hospital, School of Medicine, Zhejiang University, Hangzhou, China; ^2^Brain Research Institute, Zhejiang University, Hangzhou, China; ^3^Collaborative Innovation Center for Brain Science, Zhejiang University, Hangzhou, China

**Keywords:** intracerebral hemorrhage (ICH), necroptosis, microglia, melatonin, A20

## Abstract

Cell death is deeply involved in pathophysiology of brain injury after intracerebral hemorrhage (ICH). Necroptosis, one of the recently discovered forms of cell death, plays an important role in various diseases, including ICH. Previous studies have suggested that a considerable number of neurons undergoes necroptosis after ICH. However, necroptosis of microglia after ICH has not been reported to date. The present study demonstrated for the first time that necroptosis occurred in the microglia surrounding the hematoma after ICH in C57 mice, and melatonin, a hormone that is predominantly synthesized in and secreted from the pineal gland, exerted a neuroprotective effect by suppressing this process. When we further explored the potential underlying mechanism, we found that melatonin inhibits RIP3-mediated necroptosis by regulating the deubiquitinating enzyme A20 (also known as TNFAIP3) expression after ICH. In summary, we have demonstrated the role of microglial necroptosis in the pathogenesis of ICH. More importantly, A20 was identified as a novel target of melatonin, which opens perspectives for future research.

## Introduction

Intracerebral hemorrhage (ICH) is a significant cause of morbidity and mortality worldwide. As one of the most serious forms of stroke, ICH affects ~2 million people worldwide each year (1–3). Primary injuries after ICH are usually caused by the mechanical damage of the hematoma to the surrounding brain tissues. Following the primary injuries, secondary injuries, including inflammation and cell death, are extensively involved in the pathological processes following hemorrhagic events (4). Treatment of secondary injuries is one of the important interventions after ICH.

Cell death is a hallmark of secondary brain injury after ICH (5). Numerous experimental and clinical observations indicate a variety of cell death forms and mechanisms take place during hemorrhagic stroke (6). Among them, programmed cell death is highly correlated with the homeostatic mechanisms of the nervous system. Necroptosis, also known as programmed necrosis, shares upstream signaling elements with apoptosis, such as tumor necrosis factor (TNF), Fas-associated death domain (FADD), and Toll-like receptor (TLR) (7). However, apoptosis and necroptosis have distinct outcomes.

Apoptotic cells usually induce a non-inflammatory response, whereas necroptosis triggers an inflammatory response (8) due to the rapid loss of plasma membrane integrity prior to the exposure of phagocytic signal (9), which in turn causes the release of intracellular damage-associated molecular patterns (10), which induce inflammation. Microglia, which are primary immune cells of the central nervous system (CNS), undergo necroptosis in various pathological processes, such as ischemic stroke (11), retinal degeneration (12), and spinal cord injury (12). Therefore, it may be beneficial to inhibit necroptosis to reduce neuroinflammation and improve neuronal survival in the context of disease, particularly in microglia (13).

Melatonin (N-acetyl-5-methoxytryptamine) is a hormone that is predominantly synthesized in and secreted from the pineal gland. (14) In 1958, Lerner et al. were the first to isolate melatonin from bovine pineal gland extracts and named it according to its ability to aggregate melanin granules (15). Initial research on melatonin was focused on its regulation of circadian and circannual cycles (16). Later, the multifunctional roles of melatonin were further explored. Melatonin serves as an effective antioxidant in scavenging the highly toxic hydroxyl radical and other oxygen-centered radicals (17), and it promotes the immune response (18). No serious side effects or risks have been reported in association with the ingestion of melatonin (17). These findings suggest that melatonin might play a protective role in various pathological conditions. Therapeutic effects of melatonin in various diseases, including diabetes (19), cancers (20), cardiovascular diseases (21, 22), and CNS diseases (23) have been reported.

In CNS diseases, melatonin has been shown to contribute to the maintenance of cell homeostasis and survival by regulating inflammation, apoptosis, or autophagy after different types of brain injury (24). Melatonin reportedly has a neuroprotective effect after stroke, either ischemic (25, 26) or hemorrhagic (27). Melatonin has also been shown to inhibit necroptosis during myocardial ischemia and liver fibrosis (28–30), and this mechanism might explain its therapeutic effects after stroke. However, whether melatonin can inhibit necroptosis of microglia after ICH and how much protection can be achieved by inhibiting this process remain to be elucidated.

Serine/threonine kinase receptor interacting protein 1 (RIP1) and receptor-interacting protein 3 (RIP3) are important molecules in the process of necroptosis (7). A previous study revealed that RIP1 expression was significantly increased 1–3 days after ICH (31), suggesting that necroptosis occurs in the acute phase after ICH. Upon inhibition of caspase activity, especially that of caspase 8 by genetic or chemical methods, RIP1 forms a necrosome with RIP3 by interacting via their homotypic interaction motif domains and activates their kinase activities (32–34). Subsequently, the pseudokinase mixed lineage kinase domain-like protein (MLKL), is activated as an executive molecule of necroptosis (35). In turn, MLKL translocates to the plasma membrane, where it forms pores and disrupts the plasma membrane integrity (36). Although the necrosome, the characteristic complex of necroptosis, is composed of both RIP1 and RIP3, RIP3 is considered to have a more critical role than RIP1 in the process of necroptosis (37–39). Previous studies have suggested that melatonin inhibits necroptosis mainly by inhibiting the kinase activity of RIP3 (28, 29). However, the specific mechanism of RIP3 regulation by melatonin has not been elucidated.

A20, also known as TNFAIP3, is a deubiquitinating enzyme (40). A20 is widely recognized as a potent anti-inflammatory protein linked to multiple human brain autoimmune diseases, neuro-degenerative diseases, and brain tumors, as well as stroke (41–44). Voet et al. reported that A20 controls microglial activation to regulate neuroinflammation, a function closely related to inhibition of NLR family pyrin domain-containing protein 3 (NLRP3) (45). Moreover, Onizawa et al. discovered that A20 restricts RIP3-dependent necroptosis (46). Thus, we hypothesized that melatonin can upregulate the expression of A20 in the brain, especially in microglia, after ICH, which in turn inhibits the expression of RIP3, thereby exerting a protective effect by inhibiting necroptosis.

In this study, we aimed to demonstrate the occurrence of microglial necroptosis after ICH and to evaluate the effect of melatonin on this process as well as the underlying mechanism, to improve the recovery of neurological function after ICH.

## Materials and Methods

### Animals

C57 mice were purchased from SLAC Laboratory Animal Company Limited (Shanghai, China). In total, 214 male mice (8–10 weeks, 20–25 g) were used in this study. The mice were housed in a temperature- and humidity-controlled room under a standard 12-h light/dark cycle and had free access to food and water. The animal protocol was approved by the Institutional Ethics Committee of the Second Affiliated Hospital, Zhejiang University School of Medicine. The procedures were conducted according to the National Institutes of Health's Guide for the Care and the Use of Laboratory Animals and the ARRIVE (Animal Research: Reporting *in vivo* Experiments) guidelines. Experimental grouping was shown in Supplementary Figure S1.

### ICH Model

**Figure 1:**
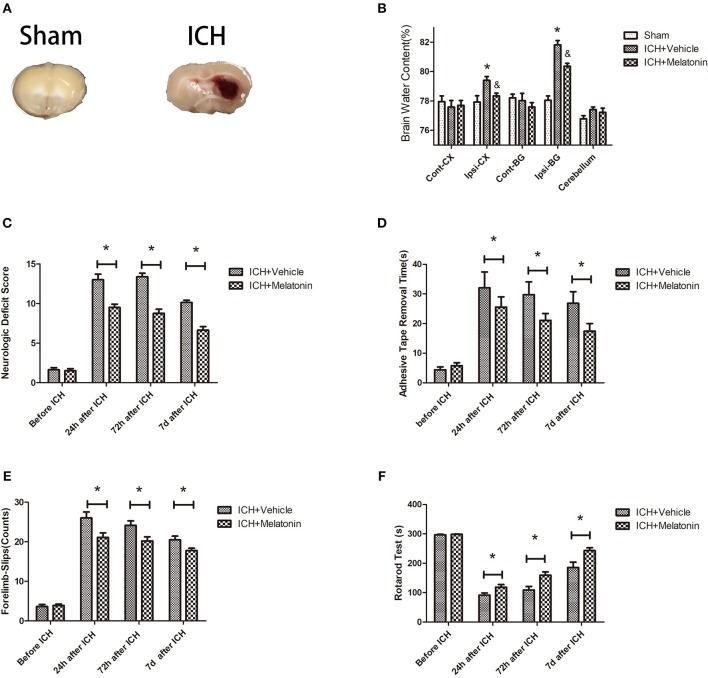
Effects of melatonin on neurologic deficit score, neurological functions, and brain edema. **(A)** Representative photographs of brain slices in the sham and ICH groups (72 h after ICH). **(B)** Quantification of brain water content at 72 h after ICH. *P <0.05 vs. sham group, ^&^*P* <0.05 vs. ICH+vehicle group (*n* = 6 in each group). **(C)** Comparison of neurologic deficit scores among ICH+vehicle and ICH+melatonin groups before ICH and at 1, 3, and 7 days after ICH. **(D)** Comparison of adhesive removal test results among the ICH+vehicle and ICH+melatonin groups before ICH and at 1, 3, 7 days after ICH. **(E)** Comparison of foot-fault test results among the ICH+vehicle and ICH+melatonin groups before ICH and at 1, 3, 7 days after ICH. **(F)** Comparison of rotarod test results among the ICH+vehicle and ICH+melatonin groups before ICH and at 1, 3, 7 days after ICH. *P <0.05.

The ICH model was established as previously described (47) (Figure 1A). Briefly, mice were anesthetized with 40 mg/kg 1% pentobarbital sodium via intraperitoneal injection. Under stereotactic guidance, a small cranial burr hole was made at a precise location (bregma coordinates: 0.5 mm anterior and 2.5 mm lateral to the midline). Autologous blood (30 μL) from the femoral artery was injected 3.5 mm deep into the right basal ganglia at a rate of 3 μL/min using a microinfusion pump, and the syringe was pulled out after 10 min.

### Drug Administration

As described previously (48, 49), melatonin (Sigma, USA) was dissolved in dimethyl sulfoxide (DMSO) and diluted with 0.9% normal saline. A dose of melatonin (20 mg/kg) or vehicle (5% DMSO) was given to mice randomly via intraperitoneal injection 30 min before ICH induction.

### Neurobehavioral Function Assessment

Four evaluation methods were used to assess the voluntary activities and motor function of mice at 24 h, 72 h, and 7 days after ICH impairment.

Neurologic function was tested before and 1, 3, 7 days after ICH by assessing body symmetry, gait, climbing, circling behavior, front limb symmetry, and compulsory circling (50). Each test result was graded from 0 to 4, with a maximum deficit score of 24.

An adhesive removal test was conducted as previously described (51). Briefly, mice were accustomed to the experimental environment for 30 min. Then, an adhesive tape strip was placed on the left hairless part of the forepaws of the mice. Mice were then put into the testing cage and the time to feel and time to remove the strip by any behavior of the mice were recorded.

For the foot-fault test, mice were individually placed on a wired grid (50 ×55 ×52 cm length/width/height) with their paws. Behavior of the mice while they were moving was recorded for 1 min. Each successful foot placement onto the bar was recorded as a step. A foot fault was recorded when a paw slipped through the grid hole. The percentage of foot faults was calculated as: 100 × faults/(successful steps + faults) (52).

A rotarod test was performed as previously described (53). Mice were placed on a Rotamex 5 apparatus (Columbus Instruments). During the test, the speed was increased from 4 rpm to 40 rpm within 5 min. After adaptation for two consecutive days, mice were tested twice daily with at least 30 min between tests. The period to fall off the rotating rod was recorded, and the data were expressed as the mean from three trials.

### Brain Water Content

We used a wet–dry method as previously described (54) to evaluate the brain water content at 72 h after ICH. Briefly, after euthanasia, mice (*n* = 6/group) were sacrificed and the brain hemispheres were collected and weighed immediately (wet weight). They were then dried at 100 °C for 48 h and weighed again to obtain the dry weight. The brain water content was calculated as follows: [(wet weight—dry weight)/(wet weight)] × 100% (55).

### Propidium Iodide (PI) Staining *in vivo*

PI staining *in vivo* to identify microglial necroptosis was conducted as previously described (31). For all experiments, PI (Beyotime, Shanghai, China) was administered intraperitoneally (10 mg/kg) 1 h before the mice were sacrificed. The brain tissue was cryoprotected by immersion in 15 and 30% sucrose solution. Then, brain sections (10 μm) were cut along the anterior–posterior lesion and placed on poly-l-lysine-coated glass slides. Sections were visualized under a fluorescence microscope (OLYMPUS BX50/BX-FLA/DP70; Olympus Co.). PI+/Iba-1+ cells were counted by observers blinded to the experimental groups. Necroptotic microglia were counted in six microscopic fields per section, and the average number in each section was calculated.

### Immunofluorescence

After the mice were anesthetized, transcardial perfusion was performed with 0.1 M PBS, followed by perfusion with 4% paraformaldehyde. The whole brain was immersed in 4% paraformaldehyde for 24 h, then cryoprotected in serial 15 and 30% sucrose solutions. The brain samples were then cut into coronal slices (9 μm) and fixed on slides. After preprocessing with 5% BSA and 0.3% triton X-100, the sections were incubated at 4°C overnight with primary antibodies, including anti-Iba-1 antibody (1:500, Abcam, Cambridge, UK, ab5079), anti-RIP1 antibody (1:200, Cell Signaling Technology, Danvers, MA, USA, CST#D94C12), anti-RIP3 antibody (1:200, Cell Signaling Technology, CST#D4G2A), anti-MLKL antibody (1:100, Santa Cruz Biotechnology, Santa Cruz, CA, USA sc-293201), anti-TNFAIP3 (A20) antibody (1:250, Abcam, ab13597). Then, the cryosections were incubated with secondary antibody [1:300, Thermo Fisher Scientific, Donkey anti-Goat IgG (H+L) Alexa Fluor 488, A32814; Donkey anti-Mouse IgG (H+L) Alexa Fluor 594, A32744; Goat anti-Rabbit IgG (H+L) Alexa Fluor 594, A-11037] at 37°C for 1 h and washed three times with PBST. Finally, the sections were observed and analyzed using a fluorescence microscope (Olympus Co., Tokyo, Japan).

### Western Blot Analysis

Brain samples around the hematoma were collected and lysed in RIPA lysis buffer (Beyotime, Shanghai, China). Western blotting was performed as previously described (56). Protein samples (40 μg/lane) were separated by 10% sodium dodecyl sulfate polyacrylamide gel and electrotransferred to polyvinylidene fluoride membranes (Millipore, Burlington, MA, USA). The membranes were blocked at room temperature for 1 h and then incubated overnight with primary antibodies, including: anti-RIP1 antibody (1:1000, Cell Signaling Technology, CST#D94C12), anti-RIP3 antibody (1:1000, Cell Signaling Technology, CST#D4G2A), anti-MLKL antibody (1:200, Santa Cruz Biotechnology, sc-293201), anti-TNFAIP3 (A20) antibody (1:1000, Abcam, ab13597), anti-RIP3 (phospho S232) antibody (1:1000, Abcam, ab195117), anti-MLKL (phosphor S345) antibody (1:1000, Abcam, ab196436), anti-NLRP3 (1:1000, Abcam, ab98151).

### Small Interfering (si)RNA and Intracerebroventricular Injection

A20 siRNA or scramble siRNA (Genomeditech, Shanghai, China) mixed with transfection reagent (Engreen Biosystem, Auckland, New Zealand) was delivered via intracerebroventricular injection as previously described (57). Briefly, the injections into the right ventricle were performed using the following coordinates relative to bregma (0.2 mm posterior, 1.0 mm lateral, and 2.0 mm deep) at 48 h prior to surgery. After the injection was completed, the needle was left in the brain for 10 min, and the burr hole was blocked with bone wax.

### Single Cell Sorting

Single cell sorting was performed as described previously (58). In brief, brain samples were fixed by transcardial perfusion with PBS before extraction. After dissociation, cells were separated by 30% Percoll density gradient separation by centrifugation at 800 × *g* for 30 min at 18°C. Then, the cells were passed through a 70-μm nylon mesh. Cell populations were sorted on an Aria SORP instrument (BD Biosciences, San Jose, CA, USA). The gating strategy is shown in Supplementary Figure S2. CD45+ (PerCP CD45, 1:200, BD Biosciences, 557235) and CD11b+ (FITC CD11b, 1:200, BD Biosciences, 557396) cells were isolated as microglia. Immediately after sorting, the cells were stored at −80°C until processed.

### Cell Lines and Coculture

The mouse microglial cell line BV2 and the mouse hippocampal cell line HT22 were cultured (37°C, 5% CO_2_) in Dulbecco's modified Eagle's medium with 10% fetal bovine serum, 100 U/ml penicillin and 100 μg/ml streptomycin. As secondary damage in ICH is mainly caused by oxidized hemoglobin, we used 100 μM OxyHb to simulate the pathological process of ICH *in vitro*, as previously described (31). Melatonin was used at 1 mM to study the effect of melatonin on necroptosis *in vitro*. A Transwell coculture system was used to investigate the effect of BV2 cells on HT22 cells. BV2 cells were cultured at a density of 5 ×10^4^ in Transwell inserts (pore size 0.4 μm; Corning, Corning, NY, USA) placed above the HT22 neuronal layer.

### Cell Viability Assay and Cytotoxicity Assay

As necroptosis is a type of cell death, cell viability and cytotoxicity can reflect its occurrence *in vitro*. Cells were cultured in 96-well plates. The CCK-8 cell counting kit (Beyotime, Shanghai, China) was used to evaluate cell viability according to the manufacturer's instructions. Twenty microliters of CCK-8 solution was added to 200 μl of cell culture medium, after which the plates were incubated at 37°C for 2 h. Then, the absorbance at 450 nm was measured. Cytotoxicity was evaluated using an LDH lactate dehydrogenase cytotoxicity test kit (Beyotime). Briefly, cells were cultured in serum-free medium for 24 h. Then, the plates were centrifuged at 400 × *g* for 5 min. One hundred twenty microliters of supernatant of each well was transferred to a new 96-well plate, and the absorbance at 490 nm was measured immediately.

### ELISA

To explore changes in inflammatory factors, a TNF ELISA KIT (Abcam, ab100747) was used according to the manufacturer's instructions to quantify the levels of TNF in BV2 cell supernatant.

### Reactive Oxygen Species (ROS) Assay

ROS can be both an inducer and a product of necroptosis. ROS levels in cells were examined using a ROS Assay Kit (JianCheng, Nanjing, China) according to the manufacturer's instructions. In brief, cells were trypsinized, collected, and incubated with 10 μM 2′,7′-dichlorodihydrofluorescein diacetate at room temperature for 30 min. After two washes, intracellular ROS production was measured by fluorescence detection using a microplate reader at an excitation wavelength of 485 nm and an emission wavelength of 535 nm. Protein levels in the cells were measured using a detergent-compatible protein assay kit (Bio-Rad, Hercules, CA, USA). ROS levels are reported as fluorescence/mg protein.

### Annexin V and PI Staining *in vitro*

Cells were cultured in six-well plates and subjected to melatonin treatment. Cells were trypsinized with 0.25% trypsin (without EDTA) and centrifuged at 1000 × *g* for 5 min and resuspended in 300 μl of binding buffer. Subsequently, 1 μl of Annexin V and 1 μl of PI (Becton Dickinson, Franklin Lanes, NJ, USA) were added to the cell suspension. After 30 min of incubation at 37°C in the dark, the cells were analyzed by flow cytometry (FACSCalibur; BD Biosciences, San Diego, CA, USA). Cells were first gated based on forward and side scatter, and necroptotic cells were determined as FITC+/PI+.

### Measurement of Mitochondrial Membrane Potential (Δψm)

BV2 cells were cultured in a six-well plate. The Δψ*m* was measured using a JC-1 kit (Beyotime, Shanghai, China) following the manufacturer's instructions. After rinsing with PBS, cells were incubated with JC-1 staining solution at 37°C for 20 min, then washed with JC-1 staining buffer. Then, the cells were imaged using a fluorescence microscope (Olympus, Tokyo, Japan). The fluorescence intensity was measured using a fluorometric microplate reader (FilterMax F5, Molecular Devices, Sunnyvale, USA) at dual wavelengths: excitation and emission at 485 and 530 nm (to detect JC-1 monomer) and at 530 and 590 nm (to detect JC-1 polymer).

### Transmission Electron Microscopy

Mice were sacrificed and perfused with 0.9% saline and 4% PFA. Fragments of peri-hematoma tissues (~1 mm^3^) were collected and immersed in glutaraldehyde (2.5%) at 4°C overnight. The tissues were preprocessed as previously described (59, 60). The samples were sliced into 100-nm sections and stained with 4% uranyl acetate and 0.5% lead citrate. The ultrastructure of the tissues was evaluated by transmission electron microscopy (Philips Tecnai 10, Netherlands).

### Statistical Analysis

All data are expressed as the mean ± standard deviation (SD) and were analyzed in GraphPad Prism v. 6.0. Data were tested for normality of distribution by the Kolmogorov-Smirnov test. One-way ANOVA was applied for comparing multiple groups. The analyses were conducted in SPSS v. 22.0 (SPSS Inc.). Statistical significance was defined as *P* <0.05.

## Results

### Melatonin Reduces Brain Edema, Improves Neurological and Motor Function After ICH

We evaluated the brain water content to explore the effects of melatonin treatment on ICH-induced brain edema. No significant differences were noted in the contralateral cortex, contralateral basal ganglia, or cerebellum between sham, ICH+vehicle, and ICH+melatonin groups. However, compared to the sham group, the brain water content was increased in the ipsilateral cortex and ipsilateral basal ganglia of mice in the ICH+vehicle group, whereas melatonin treatment significantly reduced the brain water content (Figure 1B). To explore the effect of melatonin on the neurological function of mice after ICH, we carried out various neurological tests before ICH and at 24 h, 72 h, and 7 days after ICH. Melatonin or vehicle (5% DMSO) was given to mice randomly via intraperitoneal injection 30 min before ICH induction. The neurological deficit scores were significantly increased after ICH and peaked at 72 h, whereas melatonin significantly improved neurological function (Figure 1C). The results of motor function assessment showed trends similar to those of neurological deficit scores (Figures 1D–F).

### Microglia Undergo Necroptosis After ICH

**Figure 2:**
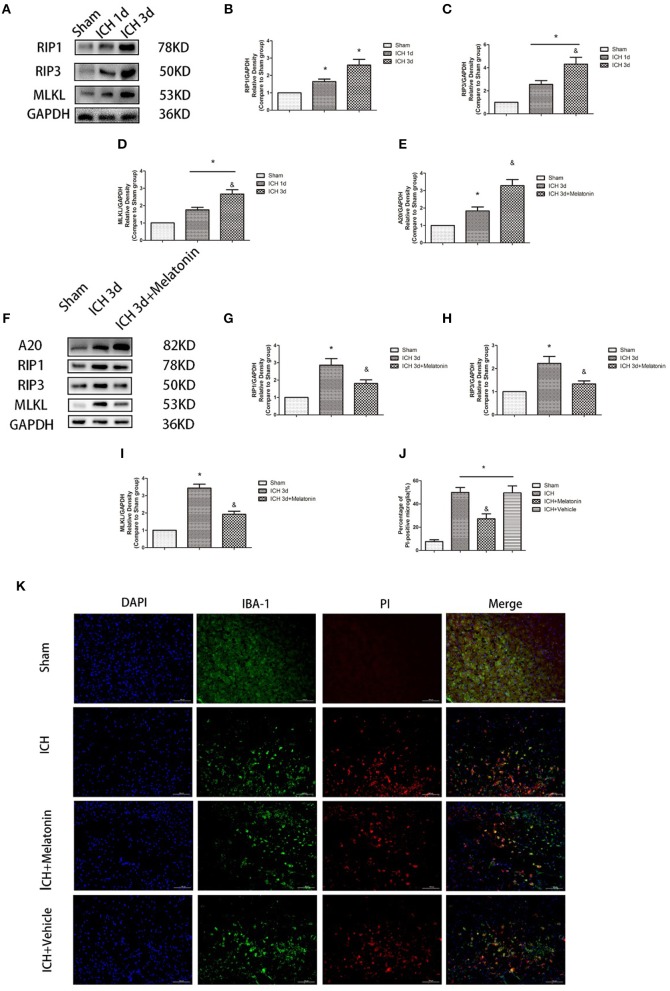
Melatonin suppresses necroptosis in microglia after ICH. **(A)** RIP1, RIP3, and MLKL protein expression was significantly enhanced at 1 and 3 days after ICH. **(B)** RIP1 expression, *P <0.05 vs. sham group (*n* = 6/group). **(C)** RIP3 expression. *P <0.05 vs. sham group (*n* = 6/group), ^&^*P* <0.05 vs. ICH 1-day group (*n* = 6/group). **(D)** MLKL expression. *P <0.05 (*n* = 6 in each group), ^&^*P* <0.05 vs. ICH 1-day group (*n* = 6/group). **(E,K)** PI staining around ICH hematoma. A significant increase in PI+ microglia was observed in the ICH group. *P <0.05 vs. sham group (*n* = 6/group). Melatonin treatment significantly decreased PI+ microglia compared with the levels in the ICH+vehicle group. ^&^*P* <0.05 vs. ICH+vehicle group (*n* = 6/group). **(F)** Expression of A20, RIP1, RIP3, and MLKL proteins in sorted microglia of each group. **(G)** A20 expression. **(H)** RIP1 expression. **(I)** RIP3 expression. **(J)** MLKL expression. *P <0.05 vs. sham group (*n* = 6/group), ^&^*P* <0.05 vs. ICH 3-day group (*n* = 6/group).

**Figure 3:**
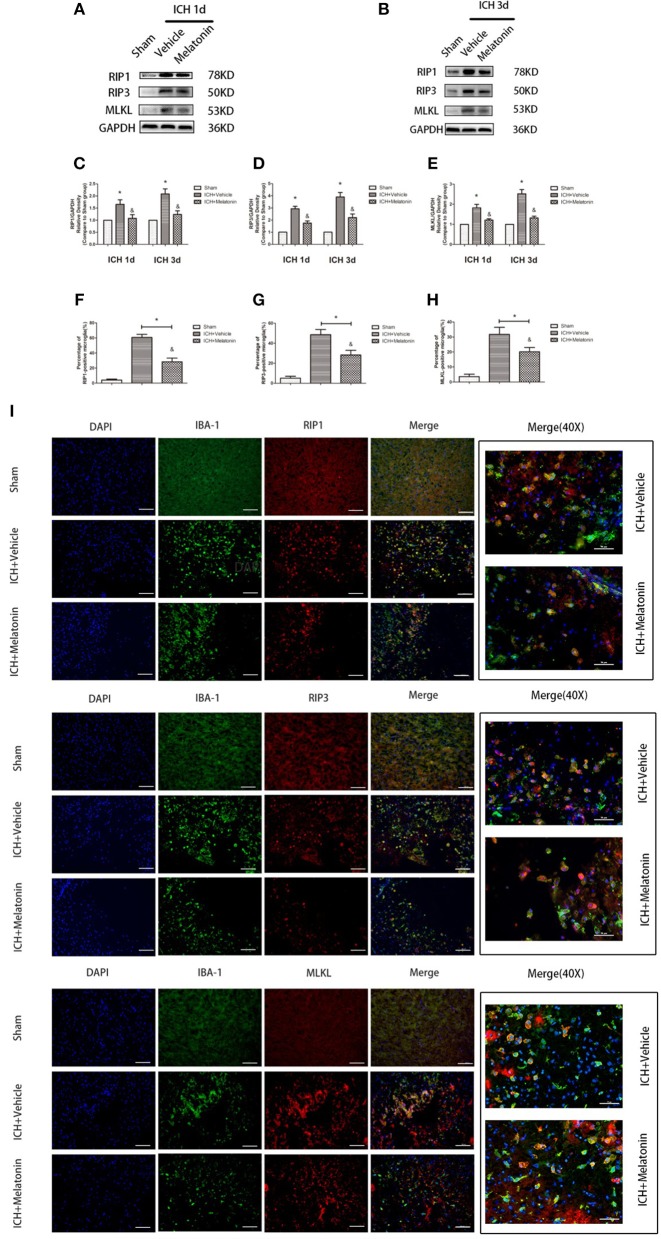
Melatonin suppresses microglial necroptosis at the early stage after ICH. **(A,B)** Western blots for RIP1, RIP3, and MLKL proteins in mice pretreated or not with melatonin via intraperitoneal injection, at 1 day and 3 days after ICH. **(C)** RIP1 expression. *P <0.05 vs. corresponding sham group (*n* = 6/group), ^&^*P* <0.05 vs. corresponding ICH+vehicle group (*n* = 6/group). **(D)** RIP3 expression. *P <0.05 vs. corresponding sham group (*n* = 6/group), ^&^*P* <0.05 vs. corresponding ICH+vehicle group. **(E)** MLKL expression. *P <0.05 vs. corresponding sham group (*n* = 6/group), ^&^*P* <0.05 vs. corresponding ICH+vehicle group. **(F–I)** Immunofluorescence staining showing the distribution of RIP1/RIP3/MLKL+ microglia surrounding hematoma at 3 days after ICH. Corresponding individually stained fluorescence images of higher magnification (40×) are shown in Supplementary Figure S3. *P <0.05 vs. sham group (*n* = 6/group), ^&^*P* <0.05 vs. ICH+vehicle group (*n* = 6/group).

To explore the involvement of necroptosis of microglia after ICH, we performed a series of experiments. The expression of RIP1, RIP3, MLKL, which are executive molecules in necroptosis, was significantly increased at 24 h and 72 h after ICH (*P* <0.05 vs. sham group; Figures 2A–D). Correspondingly, cells that were costained for these molecules and Iba-1 were also remarkably increased at 72 h after ICH (*P* <0.05 vs. sham group; Figures 3F–I). To further illustrate the occurrence of microglial necroptosis after ICH, we isolated microglia, and we found that at 3 days after ICH, the expression of necroptosis-related proteins (RIP1, RIP3, MLKL) in microglia was significantly higher than that in sham group (*P* <0.05 vs. sham group; Figures 2F–I). We also counted PI+ microglia in frozen brain sections at 72 h after ICH. PI+/Iba-1+ cells were remarkably increased in brain tissues surrounding hematomas after ICH (*P* <0.05 vs. sham group; Figures 2J,K). These results demonstrated that necroptosis occurred in microglia surrounding the hematoma after ICH.

### Melatonin Plays a Protective Role by Suppressing Necroptosis in Microglia

To explore the effects of melatonin on necroptosis after ICH, the protein levels of RIP1, RIP3 and MLKL were detected by western blot analysis at 24 h and 72 h after ICH. As shown in Figures 3A–E, the expression of these proteins was significantly increased in the ICH+vehicle group compared to the sham group (*P* <0.05), whereas melatonin treatment significantly suppressed the increases (*P* <0.05 vs. ICH+vehicle group). Immunofluorescence staining revealed that cells costained for these molecules and Iba-1 were decreased in the ICH+melatonin compared to the ICH+vehicle group (*P* <0.05; Figures 3F–I). Further, melatonin significantly reduced the expression of necroptosis-related proteins (RIP1, RIP3, MLKL) at 3 days after ICH in microglia (*P* <0.05 vs. ICH 3-day group; Figures 2F–I). These results indicated that melatonin suppresses necroptosis of microglia in brain tissues after ICH.

### Melatonin Reduces Mitochondrial Damage in Microglia After ICH

**Figure 4:**
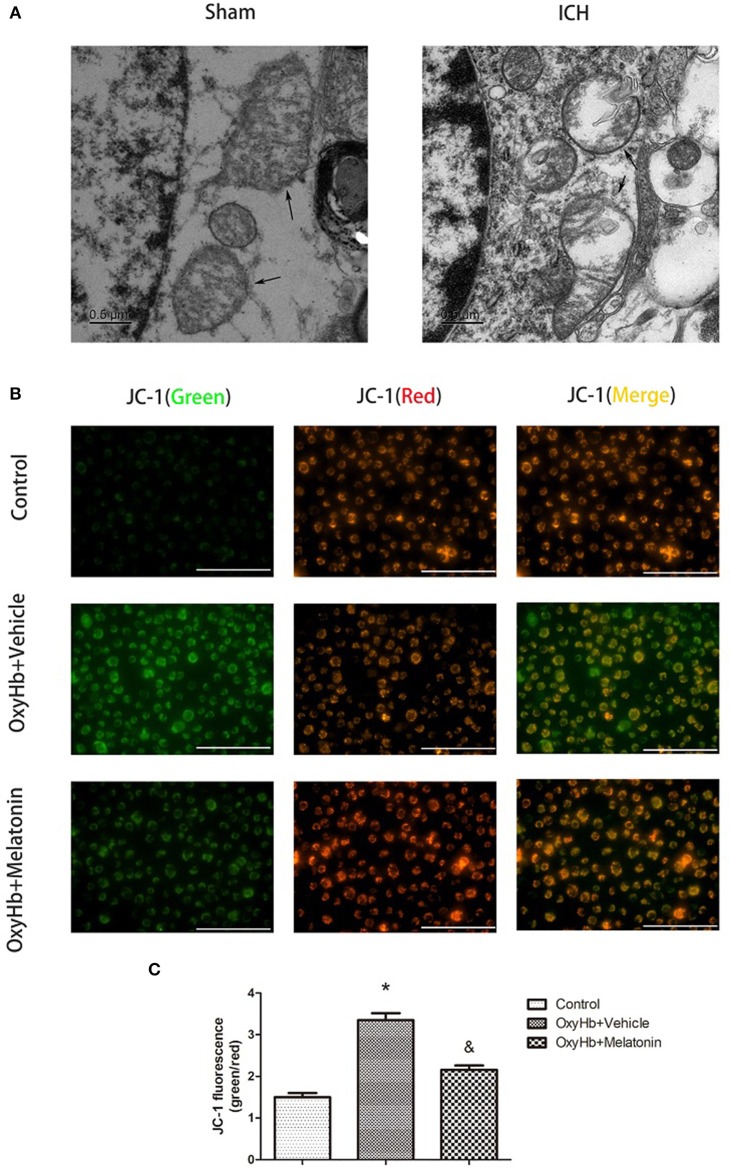
Mitochondrial damage after ICH. **(A)** TEM images of mitochondrial ultrastructure (black arrow) in basal ganglion area. **(B)** Representative photographs of JC-1-staied BV2 cells. **(C)** Quantification of JC-1 fluorescence intensity using a fluorometric microplate reader at dual wavelengths. Treatment with 10 μM OxyHb significantly decreased the Δψm of BV2 cells, whereas pre-incubation with melatonin attenuated the OxyHb-induced collapse of the Δψm. *P <0.05 vs. control group (*n* = 6 in each group), ^&^*P* <0.05 vs. OxyHb+vehicle group (*n* = 6/group).

Mitochondrial damage has been shown to be involved in necroptosis (8, 61). Therefore, we used TEM to detect mitochondrial damage at 72 h after ICH. In the brain tissues of the ICH group, mitochondria with obvious vacuolization and swelling were observed (Figure 4A). Next, we determined the Δψ*m* of BV2 cells *in vitro* by JC-1 staining. As shown in Figure 4B, the green fluorescence intensity was increased in OxyHb+vehicle group compared to the sham group, indicating a decline of Δψ*m*; however, pre-incubation with melatonin attenuated the OxyHb-induced collapse of the Δψ*m* (*P* <0.05). A microplate fluorescence read-out yielded similar results (Figure 4C). These results suggested that melatonin reduces mitochondrial damage after ICH by suppressing necroptosis.

### Melatonin Prevents Cytotoxic and ROS Production-Enhancing Effects of OxyHb *in vitro*

**Figure 5:**
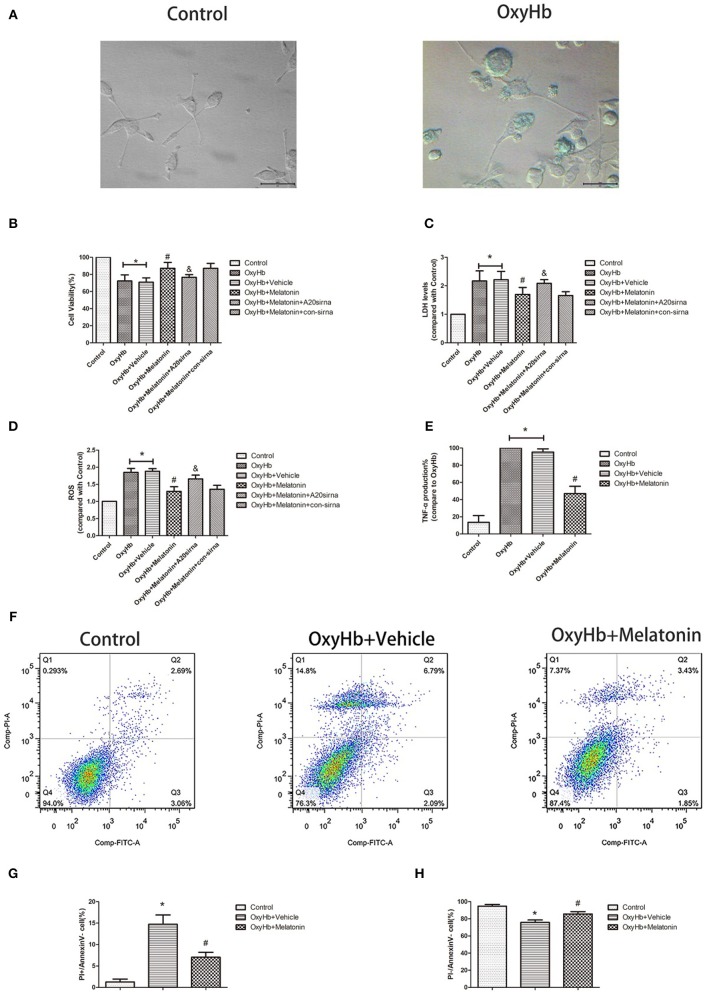
OxyHb decreases cell viability, exerts cytotoxicity, and promotes inflammatory factor production in BV2 cells, thus inducing necroptosis in HT22 cells. Pre-incubation with melatonin effectively reduces the occurrence of these effects. **(A)** Representative photographs of BV2 cells in the control and OxyHb group (72 h). BV2 cells in the OxyHb group showed cell swelling and plasma membrane rupture. **(B)** Results of cell viability assay. **(C)** Result of LDH assay. **(D)** Results of ROS production assay. **(E)** Results of TNF production assay in BV2 cell supernatant. OxyHb increased TNF production in BV2 cells, whereas melatonin significantly suppressed this. **(F–H)** Flow-cytometric analysis of HT22 cells cocultured with BV2 cells exposed to different treatments. HT22 cells cocultured with BV2 cells treated with OxyHb showed a higher necroptotic population (defined as PI+/FITC−), whereas melatonin significantly reduced the necroptotic population. *P <0.05 vs. control group (*n* = 6/group), ^#^*P* <0.05 vs. OxyHb+vehicle group (*n* = 6/group), ^&^*P* <0.05 vs. OxyHb+melatonin+con-siRNA group.

Figure 5A shows representative photographs of OxyHb-treated and control BV2 cells. Swollen cells with ruptured plasma membrane can be observed in the OxyHb-treated group. We examined the effects of melatonin on necroptosis *in vitro* by cell viability and cytotoxicity assays. As shown in Figures 5B,C, cell viability was decreased in the OxyHb+vehicle group as compared to the control group (*P* <0.05), whereas pretreatment with melatonin significantly improved cell viability (*P* <0.05). The cytotoxicity assays suggested that OxyHb had a cytotoxic effect on BV2 cells (*P* <0.05), which was prevented by melatonin pretreatment (*P* <0.05). ROS were measured to investigate the effects of melatonin on ROS production in BV2 cells. As shown in Figure 5D, treatment with OxyHb remarkably promoted ROS production, which was suppressed by pretreatment with melatonin (*P* <0.05). The above results indicated that melatonin improves BV2 cell viability and reduces cytotoxicity and ROS generation *in vitro*.

### Melatonin Suppresses TNF Secretion by Microglia, Thus Reducing Neuronal Necroptosis

As the main immune cells in the nervous system, changes in microglia would affect the homeostasis of other cells, such as neurons. We firstly explored whether the inflammatory factor TNF, a common inducer of necroptosis, is involved in this process. The concentration of TNF was significantly higher in the culture medium of BV2 cells treated with OxyHb than that in that of the control group, whereas melatonin pretreatment significantly reduced TNF production (Figure 5E, *P* <0.05). Next, we explored the effects of necroptotic microglia on neurons after ICH by using a Transwell system. HT-22 neurons were cocultured with BV2 cells exposed to various treatments. The neurons were then stained with Annexin V and PI and analyzed by flow cytometry. The necroptotic population (defined as Annexin V–/PI+) was increased in neurons cocultured with BV2 cells treated with OxyHb when compared with the control group, whereas pretreatment with melatonin suppressed this increase (Figures 5F–H; *P* <0.05). These results indicated that melatonin suppresses TNF secretion by reducing microglial necroptosis, which in turn reduces neuronal necroptosis.

### A20 Is Expressed in the Acute Phase in Microglia After ICH, and Melatonin Promotes Its Expression and Thus Plays a Protective Role

**Figure 6:**
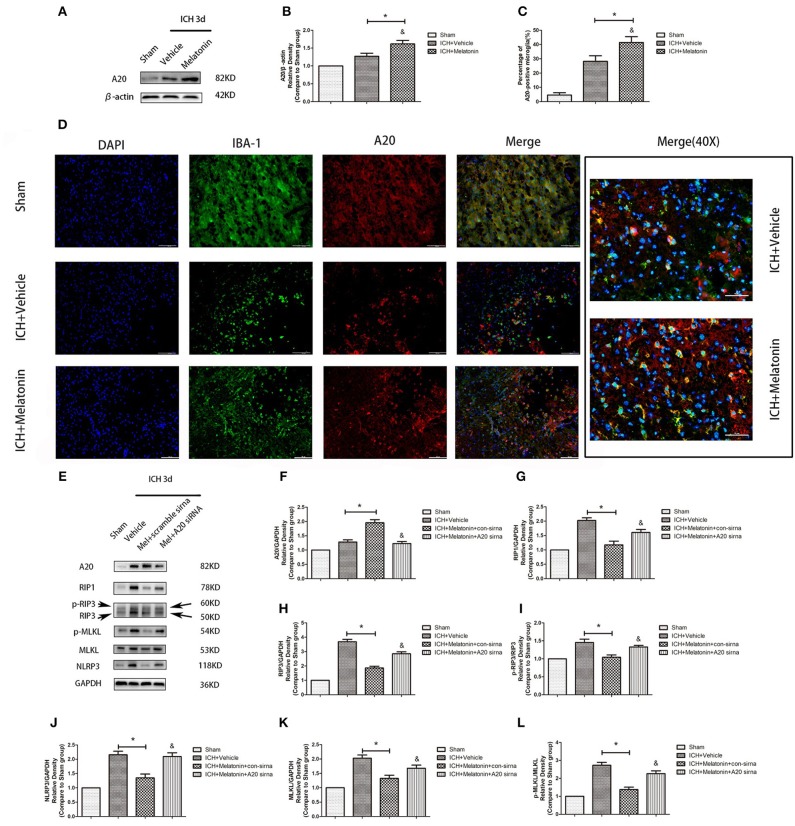
Role of A20 in the regulation of necroptosis after ICH by melatonin. **(A)** Western blots for A20 proteins in mice pretreated or not with melatonin via intraperitoneal injection, at 3 day after ICH. **(B)** A20 expression. *P <0.05 vs. sham group (*n* = 6/group), ^&^*P* <0.05 vs. ICH+vehicle group (*n* = 6/group). **(C,D)** Immunofluorescence staining showing the distribution of A20+ microglia surrounding hematoma at 72 h after ICH. A significant increase in A20+/Iba-1+ cells was observed in the ICH+vehicle group. *P <0.05 vs. sham group (*n* = 6/group). Melatonin treatment significantly enhanced the increase in A20+/Iba-1+ cells. ^&^*P* <0.05 vs. ICH+vehicle group (*n* = 6/group). Corresponding individually stained fluorescence images of higher magnification (40×) are shown in Supplementary Figure S3. **(E)** A20 knockdown attenuated the inhibitory effects of melatonin on microglial necroptosis. **(F)** A20 expression, **(G)** RIP1 expression, **(H)** RIP3 expression, **(I)** p-RIP3 expression, **(J)** NLRP3 expression, **(K)** MLKL expression, and **(L)** p-MLKL expression. *P <0.05 vs. sham group (*n* = 6/group), ^&^*P* <0.05 vs. ICH+melatonin+con-siRNA group.

We analyzed the expression of A20 at 72 h after ICH; A20 expression was significantly increased after ICH (Figures 6A,B; *P* <0.05 vs. sham group). Treatment with melatonin significantly enhanced the expression of A20 after ICH (Figures 6A,B; *P* <0.05 vs. ICH+vehicle group). Accordingly, immunofluorescence analysis revealed that A20 was expressed in the microglia surrounding the hematoma after ICH, and A20 expression was significantly increased after treatment with melatonin (Figures 6C,D, *P* <0.05). To verify the above results, we measured A20 expression in microglia at 3 days after ICH. The results showed that A20 expression was significantly enhanced when compared with the sham group (Figures 2E,F; *P* <0.05 vs. sham group). In line with the above results, melatonin treatment remarkably increased A20 expression after ICH (Figures 2E,F; *P* <0.05 vs. ICH 3-day group). We investigated the relationship between A20 and RIP3-dependent necroptosis after ICH further by using A20 siRNA. As shown in Figure 6E, A20 siRNA transfection blocked the activating effect of melatonin on A20 expression (*P* <0.05 vs. ICH+melatonin+con-siRNA group). Moreover, A20 siRNA blocked the effects of melatonin on RIP1, RIP3, MLKL expression (*P* <0.05 vs. ICH+melatonin+con-siRNA group). Melatonin reduced the phosphorylation of RIP3 and MLKL, which was prevented by A20 siRNA transfection (Figures 6E–L; *P* <0.05). *In vitro*, A20 siRNA abolished the effects of melatonin on viability and ROS production in BV2 cells (Figures 5B–D). As A20 is generally considered to be a negative regulator of NLRP3, we also detected NLRP3 expression. The results showed that A20 siNRA lowered the promotive effect of melatonin on NLRP3 expression (Figures 6E–L; *P* <0.05). These results indicated that melatonin inhibits RIP3-mediated necrotic apoptosis following ICH by regulating A20 expression.

## Discussion

**Figure 7:**
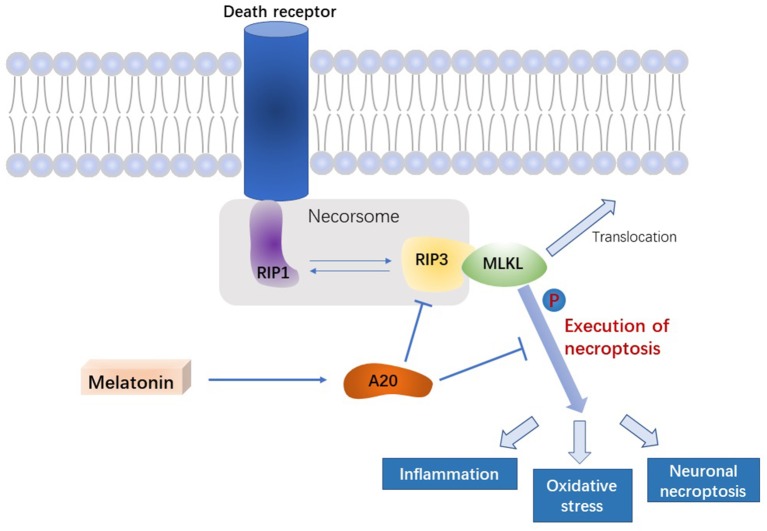
Signal pathway underlying the effect of melatonin on microglial necroptosis after ICH. After ICH, by upregulating A20 protein expression, melatonin suppresses RIP3 phosphorylation in microglia, which in turn inhibits the activation of MLKL, the executive molecule of necroptosis, thereby inhibiting inflammation, reducing mitochondrial damage, and reducing oxidative stress, ultimately playing a neuroprotective role after ICH.

In this study, we demonstrated that melatonin inhibits necroptosis of microglia after ICH, thereby reducing brain edema and improving neurological function in the acute phase of ICH. In addition, we uncovered the potential underlying mechanism, in which melatonin regulates A20, thus affecting RIP3-dependent necroptosis (Figure 7).

Increasing evidence indicates that necroptosis plays a crucial role in the pathogenesis of several diseases with an inflammatory component (13). Shen et al. (31) found that necroptosis is an important mechanism of neuronal death after ICH, and treatment with necrostatin-1, a specific inhibitor of RIP1, could rescue the neurons from necroptosis, revealing a potential therapeutic target for ICH. Microglia are associated with necroptosis in different disease models (62–64); however, the exact underlying mechanism remained unclear. Moreover, there are no reports on microglial necroptosis after ICH. This study for the first time demonstrated that a significant amount of microglia around the hematoma did undergo necroptosis around 24 and 72 h after ICH. Although the relationship between necroptosis and inflammation has not been fully elucidated, necroptosis is generally thought to induce inflammation as, unlike apoptosis, it is essentially a type of necrotic cell death. In addition to inflammation, necroptosis causes some other cascade reactions, including mitochondrial damage, which exacerbates the release of ROS (32), which induce necroptosis. In line with previous experimental results (65), the present study suggested that mitochondrial damage and ROS accumulation after ICH might be caused by necroptosis. Therefore, necroptosis can play an intermediate role in the induction of inflammation by microglia after ICH. As resident macrophages in the CNS, microglia exert a variety of neuroprotective functions, such as phagocytosis and elimination, secretion of anti-inflammatory factors, growth factors, and promotion of the recovery of neurological function (66, 67). Thus, although microglia account for only approximately 10% of cells in the CNS, their death after ICH aggravates outcome. Therefore, we hypothesized that necroptosis plays an important role in the pathological processes after ICH.

A previous study revealed that A20 expression was increased after ICH and was associated with anti-inflammatory effects by inhibiting NF-κB activation (44). In addition to its NF-κB-regulatory function, A20 is a ubiquitin-regulating enzyme, and increasing evidence shows that the central importance of its functions can be attributed to its ability to modulate ubiquitin-dependent signaling cascades (42). Ubiquitination is one of most prevalent post-translational protein modifications that regulate a variety of important physiological functions. During ubiquitination, ubiquitin, a highly conserved 76-amino-acid protein, is conjugated to lysine residues of diverse cellular proteins (66, 68). The E3 ubiquitin ligase activity of A20 has been shown to directly ubiquitinate RIP1, thus triggering proteasomal degradation of RIP1, which in turn inhibits TNF-induced NF-κB activation (69). Therefore, in the present study, we mainly focused on the role of A20 as a deubiquitinating enzyme. As RIP1 is involved in apoptosis, it seems plausible that A20 could inhibit cellular apoptosis by promoting RIP1 degradation. However, a previous studies indicated that its anti-apoptotic effect is cell type- and stimulus-specific; thus, its protective effect is controversial (70). One study reported that A20 tends to inhibit apoptosis via various mechanisms (41); however, other results indicated that in certain pathological conditions, such as IBD, A20 binds to RIP1 to enhance TNF-induced apoptosis (71). We did not further explore the effect of A20 on microglial apoptosis in this study. However, regardless of promotive or inhibitory effects, A20 is considered to directly interact with RIP1 rather than via regulating NF-κB (71). As mentioned earlier, the necrosome is composed of both RIP1 and RIP3. Because RIP1 is involved in various biological processes, RIP3 is more valuable than RIP1 when studying necroptosis specifically. Moreover, RIP1 is not considered to be ubiquitinated in the necrosome, which suggests that A20 might regulate necroptosis by regulating RIP3. Furthermore, RIP3 has been regarded a specific activator of necroptosis because it regulates RIP1 phosphorylation (32) and switches TNF-induced cell death from apoptosis to necroptosis (33). The potential of A20 to suppress TNF-induced necroptosis by affecting RIP3 was observed in L929 cells and in primary mouse T cells (46, 72). In line herewith, in the present study, A20 regulated RIP3, thus inhibiting necroptosis.

Melatonin intervenes in different pathological processes through various mechanisms, e.g., it reduces inflammation (24, 73, 74), protects mitochondrial oxidoreductase and superoxide dismutase, thus improving ROS scavenging (75), and inhibits apoptosis (24, 76). In myocardial ischemia, melatonin inhibits necroptosis in cardiomyocytes by inhibiting RIP3 (29). In our study, melatonin inhibited necroptosis of microglia after ICH. We further explored the possible underlying mechanism. A20 is a regulatory protein of the NF-κB signaling pathway, with effects quite similar to those observed after treatment with melatonin. Accordingly, we found that melatonin inhibits RIP3-mediated necroptosis by promoting A20 expression. Interestingly, the NLRP3-regulatory effect of melatonin could be abolished by A20 siRNA transfection *in vitro*. This opens a new perspective for future exploration of NLRP3-mediated inflammation.

This study had some limitations that cannot be ignored. Firstly, melatonin might exert its neuroprotective effects after ICH by regulating various functions; the present study only explored its effect on necroptosis. Secondly, we only used cell lines for investigating the effect of melatonin on necroptosis *in vitro*; considering some subtle differences between cell lines and primary cells, out data would be more convincing had we used primary cells.

In conclusion, this study revealed that melatonin exerts neuroprotective effects after ICH. We demonstrated that melatonin inhibits microglial necroptosis via the A20/RIP3 pathway, in turn reducing cell death, inflammation, and mitochondrial damage. These findings implicate A20 as a novel potential therapeutic target for ICH.

## Data Availability

The datasets generated for this study are available on request to the corresponding author.

## Ethics Statement

The animal protocol was approved by the Institutional Ethics Committee of the Second Affiliated Hospital, Zhejiang University School of Medicine. The procedures were conducted according to the National Institutes of Health's Guide for the Care and the Use of Laboratory Animals and the ARRIVE (Animal Research: Reporting *in vivo* Experiments) guidelines.

## Author Contributions

JiaZ is the principal investigator. JL, ZS, and FL contributed to the study design, performance, and manuscript draft. YF, JinZ, SX, and SM analyzed the experimental data. WX, LS, and HW revised the manuscript and polished the language.

### Conflict of Interest Statement

The authors declare that the research was conducted in the absence of any commercial or financial relationships that could be construed as a potential conflict of interest.

## Supplementary Material

Supplementary Figure S1 Experimental grouping.jpg. *In-vivo* and *in*-*vitro* experimental groups.

Supplementary Figure S2 FACS gating strategy.jpg. Gating strategy used to isolate microglia.

Supplementary Figure S3 Single-stained fluorescence.jpg. Single-stained high-magnification (40×) fluorescence images.

## References

[1] Hemphill JC III Greenberg SM Anderson CS Becker K Bendok BR Cushman M Guidelines for the management of spontaneous intracerebral hemorrhage: a guideline for healthcare professionals froma the american heart association/american stroke association. Stroke. (2015) 46:2032–60. 10.1161/STR.0000000000000069 26022637

[2] Krishnamurthi RV Feigin VL Forouzanfar MH Mensah GA Connor M Bennett DA. Global and regional burden of first-ever ischaemic and haemorrhagic stroke during 1990–2010: findings from the Global Burden of Disease Study 2010. Lancet Global Health. (2013) 1:e259–81. 10.1016/S2214-109X(13)70089-5 25104492 PMC4181351

[3] Feigin VL Lawes CM Bennett DA Barker-Collo SL Parag V. Worldwide stroke incidence and early case fatality reported in 56 population-based studies: a systematic review. Lancet Neurol. (2009) 8:355–69. 10.1016/S1474-4422(09)70025-0 19233729

[4] Keep RF Hua Y Xi G. Intracerebral haemorrhage: mechanisms of injury and therapeutic targets. Lancet Neurol. (2012) 11:720–31. 10.1016/S1474-4422(12)70104-7 22698888 PMC3884550

[5] Chu X Wu X Feng H Zhao H Tan Y Wang L. Coupling between interleukin-1R1 and necrosome complex involves in hemin-induced neuronal necroptosis after intracranial hemorrhage. Stroke. (2018) 49:2473–82. 10.1161/STROKEAHA.117.019253 30355103

[6] Fricker M Tolkovsky AM Borutaite V Coleman M Brown GC. Neuronal cell death. Physiol Rev. (2018) 98:813–80. 10.1152/physrev.00011.2017 29488822 PMC5966715

[7] Linkermann A Green DR. Necroptosis. N Engl J Med. (2014) 370:455–65. 10.1056/NEJMra1310050 24476434 PMC4035222

[8] Maeda A Fadeel B. Mitochondria released by cells undergoing TNF-alpha-induced necroptosis act as danger signals. Cell Death Dis. (2014) 5:e1312. 10.1038/cddis.2014.277 24991764 PMC4123071

[9] Golstein P Kroemer G. Cell death by necrosis: towards a molecular definition. Trends Biochem Sci. (2007) 32:37–43. 10.1016/j.tibs.2006.11.001 17141506

[10] Zhang Y Chen X Gueydan C Han J. Plasma membrane changes during programmed cell deaths. Cell Res. (2018) 28:9–21. 10.1038/cr.2017.133 29076500 PMC5752838

[11] Yang J Zhao Y Zhang L Fan H Qi C Zhang K. RIPK3/MLKL-mediated neuronal necroptosis modulates the M1/M2 polarization of microglia/macrophages in the ischemic cortex. Cerebral Cortex. (2018) 28:2622–35. 10.1093/cercor/bhy089 29746630 PMC5998990

[12] Fan H Zhang K Shan L Kuang F Chen K Zhu K. Reactive astrocytes undergo M1 microglia/macrohpages-induced necroptosis in spinal cord injury. Mol Neurodegener. (2016) 11:14. 10.1186/s13024-016-0081-8 26842216 PMC4740993

[13] Oliveira SR Amaral JD Rodrigues CMP. Mechanism and disease implications of necroptosis and neuronal inflammation. Cell Death Dis. (2018) 9:903. 10.1038/s41419-018-0872-7 30185777 PMC6125291

[14] Hu J Zhang L Yang Y Guo Y Fan Y Zhang M. Melatonin alleviates postinfarction cardiac remodeling and dysfunction by inhibiting Mst1. J Pineal Res. (2017) 62:e12368. 10.1111/jpi.12368 27696525

[15] Lerner AB Case JD Takahashi Y. Isolation of melatonin and 5-methoxyindole-3-acetic acid from bovine pineal glands. J Biol Chem. (1960) 235:1992–7. 14415935

[16] Reiter RJ. Pineal melatonin: cell biology of its synthesis and of its physiological interactions. Endocr Rev. (1991) 12:151–80. 10.1210/edrv-12-2-151 1649044

[17] Brzezinski A. Melatonin in humans. N Engl J Med. (1997) 336:186–95. 10.1056/NEJM199701163360306 8988899

[18] Maestroni GJ. The immunoneuroendocrine role of melatonin. J Pineal Res. (1993) 14:1–10. 10.1111/j.1600-079X.1993.tb00478.x 8483103

[19] Rubio-Sastre P Scheer FA Gómez-Abellán P Madrid JA Garaulet M. Acute melatonin administration in humans impairs glucose tolerance in both the morning and evening. Sleep. (2014) 37:1715–9. 10.5665/sleep.4088 25197811 PMC4173928

[20] Su SC Hsieh MJ Yang WE Chung WH Reiter RJ Yang SF. Cancer metastasis: mechanisms of inhibition by melatonin. J Pineal Res. (2017) 62:e12370. 10.1111/jpi.12370 27706852

[21] Simko F Baka T Paulis L Reiter RJ. Elevated heart rate and nondipping heart rate as potential targets for melatonin: a review. J Pineal Res. (2016) 61:127–37. 10.1111/jpi.12348 27264986

[22] Dominguez-Rodriguez A Abreu-Gonzalez P Sanchez-Sanchez JJ Kaski JC Reiter RJ. Melatonin and circadian biology in human cardiovascular disease. J Pineal Res. (2010) 49:14–22. 10.1111/j.1600-079X.2010.00773.x 20536686

[23] Pandi-Perumal SR Trakht I Srinivasan V Spence DW Maestroni GJ Zisapel N. Physiological effects of melatonin: role of melatonin receptors and signal transduction pathways. Progress Neurobiol. (2008) 85:335–53. 10.1016/j.pneurobio.2008.04.001 18571301

[24] Fernandez A Ordonez R Reiter RJ Gonzalez-Gallego J Mauriz JL. Melatonin and endoplasmic reticulum stress: relation to autophagy and apoptosis. J Pineal Res. (2015) 59:292–307. 10.1111/jpi.12264 26201382

[25] Macleod MR O'Collins T Horky LL Howells DW Donnan GA. Systematic review and meta-analysis of the efficacy of melatonin in experimental stroke. J Pineal Res. (2005) 38:35–41. 10.1111/j.1600-079X.2004.00172.x 15617535

[26] Ramos E Patiño P Reiter RJ Gil-Martín E Marco-Contelles J Parada E. Ischemic brain injury: new insights on the protective role of melatonin. Free Radical Biol Med. (2017) 104:32–53. 10.1016/j.freeradbiomed.2017.01.005 28065781

[27] Wu HJ Wu C Niu HJ Wang K Mo LJ Shao AW. Neuroprotective mechanisms of melatonin in hemorrhagic stroke. Cell Mol Neurobiol. (2017) 37:1173–85. 10.1007/s10571-017-0461-9 28132129 PMC11482116

[28] Yang Z Li C Wang Y Yang J Yin Y Liu M. Melatonin attenuates chronic pain related myocardial ischemic susceptibility through inhibiting RIP3-MLKL/CaMKII dependent necroptosis. J Mol Cell Cardiol. (2018) 125:185–94. 10.1016/j.yjmcc.2018.10.018 30365930

[29] Zhou H Li D Zhu P Ma Q Toan S Wang J. Inhibitory effect of melatonin on necroptosis via repressing the Ripk3-PGAM5-CypD-mPTP pathway attenuates cardiac microvascular ischemia-reperfusion injury. J Pineal Res. (2018) 65:e12503. 10.1111/jpi.12503 29770487

[30] Choi HS Kang JW Lee SM. Melatonin attenuates carbon tetrachloride-induced liver fibrosis via inhibition of necroptosis. Transl Res. (2015) 166:292–303. 10.1016/j.trsl.2015.04.002 25936762

[31] Shen H Liu C Zhang D Yao X Zhang K Li H. Role for RIP1 in mediating necroptosis in experimental intracerebral hemorrhage model both *in vivo* and *in vitro*. Cell Death Dis. (2017) 8:e2641. 10.1038/cddis.2017.58 28252651 PMC5386555

[32] Cho YS Challa S Moquin D Genga R Ray TD Guildford M. Phosphorylation-driven assembly of the RIP1-RIP3 complex regulates programmed necrosis and virus-induced inflammation. Cell. (2009) 137:1112–23. 10.1016/j.cell.2009.05.037 19524513 PMC2727676

[33] Zhang DW Shao J Lin J Zhang N Lu BJ Lin SC. RIP3, an energy metabolism regulator that switches TNF-induced cell death from apoptosis to necrosis. Science. (2009) 325:332–6. 10.1126/science.1172308 19498109

[34] He S Wang L Miao L Wang T Du F Zhao L. Receptor interacting protein kinase-3 determines cellular necrotic response to TNF-alpha. Cell. (2009) 137:1100–11. 10.1016/j.cell.2009.05.021 19524512

[35] Sun L Wang H Wang Z He S Chen S Liao D. Mixed lineage kinase domain-like protein mediates necrosis signaling downstream of RIP3 kinase. Cell. (2012) 148:213–27. 10.1016/j.cell.2011.11.031 22265413

[36] Vanden Berghe T Linkermann A Jouan-Lanhouet S Walczak H Vandenabeele P. Regulated necrosis: the expanding network of non-apoptotic cell death pathways. Nat Rev. Mol Cell Biol. (2014) 15:135–47. 10.1038/nrm3737 24452471

[37] Mandal P Berger SB Pillay S Moriwaki K Huang C Guo H. RIP3 induces apoptosis independent of pronecrotic kinase activity. Mol Cell. (2014) 56:481–95. 10.1016/j.molcel.2014.10.021 25459880 PMC4512186

[38] Kaiser WJ Daley-Bauer LP Thapa RJ Mandal P Berger SB Huang C. RIP1 suppresses innate immune necrotic as well as apoptotic cell death during mammalian parturition. Proc Natl Acad Sci USA. (2014) 111:7753–8. 10.1073/pnas.1401857111 24821786 PMC4040608

[39] Kaiser WJ Sridharan H Huang C Mandal P Upton JW Gough PJ. Toll-like receptor 3-mediated necrosis via TRIF, RIP3, and MLKL. J Biol Chem. (2013) 288:31268–79. 10.1074/jbc.M113.462341 24019532 PMC3829437

[40] Boone DL Turer EE Lee EG Ahmad RC Wheeler MT Tsui C. The ubiquitin-modifying enzyme A20 is required for termination of Toll-like receptor responses. Nat Immunol. (2004) 5:1052–60. 10.1038/ni1110 15334086

[41] Abbasi A Forsberg K Bischof F. The role of the ubiquitin-editing enzyme A20 in diseases of the central nervous system and other pathological processes. Front Mol Neurosci. (2015) 8:21. 10.3389/fnmol.2015.00021 26124703 PMC4466442

[42] Catrysse L Vereecke L Beyaert R van Loo G. A20 in inflammation and autoimmunity. Trends Immunol. (2014) 35:22–31. 10.1016/j.it.2013.10.005 24246475

[43] Ma A Malynn BA. A20: linking a complex regulator of ubiquitylation to immunity and human disease. Nat Rev Immunol. (2012) 12:774–85. 10.1038/nri3313 23059429 PMC3582397

[44] Meng Z Zhao T Zhou K Zhong Q Wang Y Xiong X. A20 ameliorates intracerebral hemorrhage-induced inflammatory injury by regulating TRAF6 polyubiquitination. J Immunol. (2017) 198:820–31. 10.4049/jimmunol.1600334 27986908 PMC5220121

[45] Voet S Mc Guire C Hagemeyer N Martens A Schroeder A Wieghofer P. A20 critically controls microglia activation and inhibits inflammasome-dependent neuroinflammation. Nat Commun. (2018) 9:2036. 10.1038/s41467-018-04376-5 29789522 PMC5964249

[46] Onizawa M Oshima S Schulze-Topphoff U Oses-Prieto JA Lu T Tavares R The ubiquitin-modifying enzyme A20 restricts ubiquitination of the kinase RIPK3 and protects cells from necroptosis. Nat Immunol. (2015) 16:618–27. 10.1038/ni.3172 25939025 PMC4439357

[47] Rynkowski MA Kim GH Komotar RJ Otten ML Ducruet AF Zacharia BE. A mouse model of intracerebral hemorrhage using autologous blood infusion. Nat Protocols. (2008) 3:122–8. 10.1038/nprot.2007.513 18193028

[48] Lekic T Hartman R Rojas H Manaenko A Chen W Ayer R. Protective effect of melatonin upon neuropathology, striatal function, and memory ability after intracerebral hemorrhage in rats. J Neurotrauma. (2010) 27:627–37. 10.1089/neu.2009.1163 20350200 PMC2867555

[49] Wang Z Zhou F Dou Y Tian X Liu C Li H. Melatonin alleviates intracerebral hemorrhage-induced secondary brain injury in rats via suppressing apoptosis, inflammation, oxidative stress, DNA damage, and mitochondria injury. Transl Stroke Res. (2018) 9:74–91. 10.1007/s12975-017-0559-x 28766251 PMC5750335

[50] Wu H Wu T Han X Wan J Jiang C Chen W. Cerebroprotection by the neuronal PGE2 receptor EP2 after intracerebral hemorrhage in middle-aged mice. J Cerebral Blood Flow Metabol. (2017) 37:39–51. 10.1177/0271678X15625351 26746866 PMC5363749

[51] Bouet V Boulouard M Toutain J Divoux D Bernaudin M Schumann-Bard P. The adhesive removal test: a sensitive method to assess sensorimotor deficits in mice. Nat Protocols. (2009) 4:1560–4. 10.1038/nprot.2009.125 19798088

[52] Paes-Branco D Abreu-Villaca Y Manhaes AC Filgueiras CC. Unilateral hemispherectomy at adulthood asymmetrically affects motor performance of male Swiss mice. Experi Brain Res. (2012) 218:465–76. 10.1007/s00221-012-3034-7 22367398

[53] Liu X Liu J Zhao S Zhang H Cai W Cai M. Interleukin-4 is essential for microglia/macrophage m2 polarization and long-term recovery after cerebral ischemia. Stroke. (2016) 47:498–504. 10.1161/STROKEAHA.115.012079 26732561 PMC4729613

[54] Wu J Zhang Y Yang P Enkhjargal B Manaenko A Tang J. Recombinant osteopontin stabilizes smooth muscle cell phenotype via integrin receptor/integrin-linked Kinase/Rac-1 pathway after subarachnoid hemorrhage in rats. Stroke. (2016) 47:1319–27. 10.1161/STROKEAHA.115.011552 27006454 PMC4846549

[55] Chen M Li X Zhang X He X Lai L Liu Y Chen M. The inhibitory effect of mesenchymal stem cell on blood-brain barrier disruption following intracerebral hemorrhage in rats: contribution of TSG-6. J Neuroinflamm. (2015) 12:61. 10.1186/s12974-015-0284-x 25890011 PMC4392640

[56] Pariente R Pariente JA Rodríguez AB Espino J. Melatonin sensitizes human cervical cancer HeLa cells to cisplatin-induced cytotoxicity and apoptosis: effects on oxidative stress and DNA fragmentation. J Pineal Res. (2016) 60:55–64. 10.1111/jpi.12288 26462739

[57] Zhao L Chen S Sherchan P Ding Y Zhao W Guo Z. Recombinant CTRP9 administration attenuates neuroinflammation via activating adiponectin receptor 1 after intracerebral hemorrhage in mice. J Neuroinflam. (2018) 15:215. 10.1186/s12974-018-1256-8 30060752 PMC6066941

[58] Keren-Shaul H Spinrad A Weiner A Matcovitch-Natan O Dvir-Szternfeld R Ulland TK. A unique microglia type associated with restricting development of alzheimer's disease. Cell. (2017) 169:1276–90 e1217. 10.1016/j.cell.2017.05.018 28602351

[59] Fan LF He PY Peng YC Du QH Ma YJ Jin JX. Mdivi-1 ameliorates early brain injury after subarachnoid hemorrhage via the suppression of inflammation-related blood-brain barrier disruption and endoplasmic reticulum stress-based apoptosis. Free Radical Biol Med. (2017) 112:336–49. 10.1016/j.freeradbiomed.2017.08.003 28790012

[60] Zheng J Shi L Liang F Xu W Li T Gao L. Sirt3 Ameliorates oxidative stress and mitochondrial dysfunction after intracerebral hemorrhage in diabetic rats. Front Neurosci. (2018) 12:414. 10.3389/fnins.2018.00414 29970985 PMC6018086

[61] Matsuzawa-Ishimoto Y Shono Y Gomez LE Hubbard-Lucey VM Cammer M Neil J. Autophagy protein ATG16L1 prevents necroptosis in the intestinal epithelium. J Experi Med. (2017) 214:3687–705. 10.1084/jem.20170558 29089374 PMC5716041

[62] Kim SJ Li J. Caspase blockade induces RIP3-mediated programmed necrosis in Toll-like receptor-activated microglia. Cell Death Dis. (2013) 4:e716. 10.1038/cddis.2013.238 23846218 PMC3730412

[63] Fricker M Vilalta A Tolkovsky AM Brown GC. Caspase inhibitors protect neurons by enabling selective necroptosis of inflamed microglia. J Biol Chem. (2013) 288:9145–52. 10.1074/jbc.M112.427880 23386613 PMC3610987

[64] Huang Z Zhou T Sun X Zheng Y Cheng B Li M. Necroptosis in microglia contributes to neuroinflammation and retinal degeneration through TLR4 activation. Cell Death Different. (2018) 25:180–9. 10.1038/cdd.2017.141 28885615 PMC5729519

[65] Degterev A Huang Z Boyce M Li Y Jagtap P Mizushima N. Chemical inhibitor of nonapoptotic cell death with therapeutic potential for ischemic brain injury. Nat Chem Biol. (2005) 1:112–9. 10.1038/nchembio711 16408008

[66] Colonna M Butovsky O. Microglia function in the central nervous system during health and neurodegeneration. Ann Rev Immunol. (2017) 35:441–68. 10.1146/annurev-immunol-051116-052358 28226226 PMC8167938

[67] Ma Y Wang J Wang Y Yang GY. The biphasic function of microglia in ischemic stroke. Progress Neurobiol. (2017) 157:247–72. 10.1016/j.pneurobio.2016.01.005 26851161

[68] Hershko A Ciechanover A. The ubiquitin system. Ann Rev Biochem. (1998) 67:425–79. 10.1146/annurev.biochem.67.1.425 9759494

[69] Wertz IE O'Rourke KM Zhou H Eby M Aravind L Seshagiri S. De-ubiquitination and ubiquitin ligase domains of A20 downregulate NF-kappaB signalling. Nature. (2004) 430:694–9. 10.1038/nature02794 15258597

[70] Daniel S Arvelo MB Patel VI Longo CR Shrikhande G Shukri T. A20 protects endothelial cells from TNF-, Fas-, and NK-mediated cell death by inhibiting caspase 8 activation. Blood. (2004) 104:2376–84. 10.1182/blood-2003-02-0635 15251990

[71] Garcia-Carbonell R Wong J Kim JY Close LA Boland BS Wong TL. Elevated A20 promotes TNF-induced and RIPK1-dependent intestinal epithelial cell death. Proc Natl Acad Sci USA. (2018) 115:E9192–200. 10.1073/pnas.1810584115 30209212 PMC6166836

[72] Vanlangenakker N Vanden Berghe T Bogaert P Laukens B Zobel K Deshayes K. cIAP1 and TAK1 protect cells from TNF-induced necrosis by preventing RIP1/RIP3-dependent reactive oxygen species production. Cell Death Different. (2011) 18:656–65. 10.1038/cdd.2010.138 21052097 PMC3131911

[73] Sun CK Lee FY Kao YH Chiang HJ Sung PH Tsai TH. Systemic combined melatonin-mitochondria treatment improves acute respiratory distress syndrome in the rat. J Pineal Res. (2015) 58:137–50. 10.1111/jpi.12199 25491480

[74] Sagrillo-Fagundes L Assunção Salustiano EM Ruano R Markus RP Vaillancourt C. Melatonin modulates autophagy and inflammation protecting human placental trophoblast from hypoxia/reoxygenation. J Pineal Res. (2018) 65:e12520. 10.1111/jpi.12520 30091210

[75] Reiter RJ Mayo JC Tan DX Sainz RM Alatorre-Jimenez M Qin L. Melatonin as an antioxidant: under promises but over delivers. J Pineal Res. (2016) 61:253–78. 10.1111/jpi.12360 27500468

[76] Hosseinzadeh A Kamrava SK Joghataei MT Darabi R Shakeri-Zadeh A Shahriari M. Apoptosis signaling pathways in osteoarthritis and possible protective role of melatonin. J Pineal Res. (2016) 61:411–25. 10.1111/jpi.12362 27555371

