# Supplementary figures and images for: Melatonin Suppresses Microglial Necroptosis by Regulating Deubiquitinating Enzyme A20 After Intracerebral Hemorrhage
Source: Front Immunol. 2019 Jun 14;10:1360. doi: 10.3389/fimmu.2019.01360 (PMC6587666; doi:10.3389/fimmu.2019.01360)

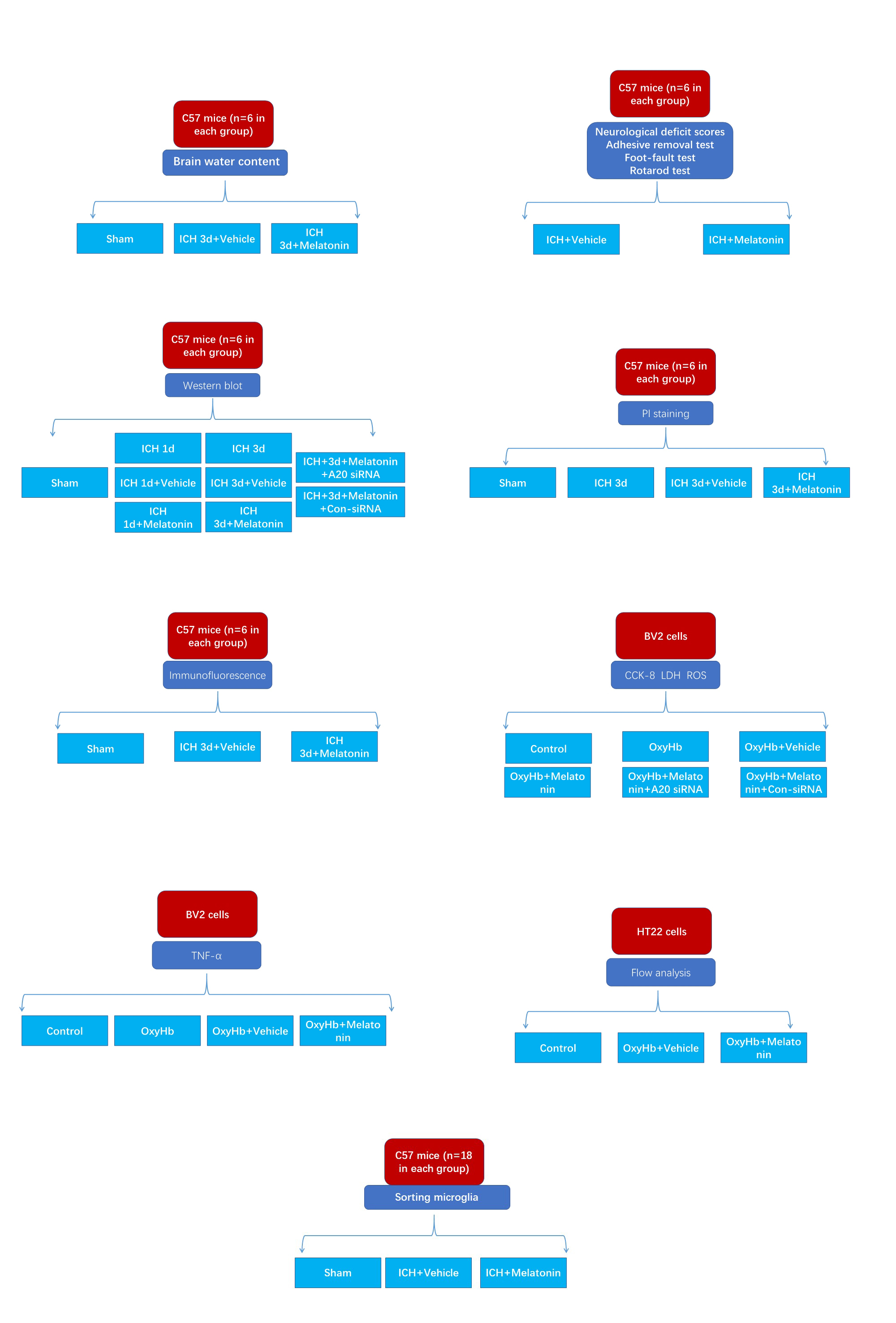

Supplement: Supplementary Figure S1 — Experimental grouping.jpg. In-vivo and in-vitro experimental groups. [file Image_1.JPEG]

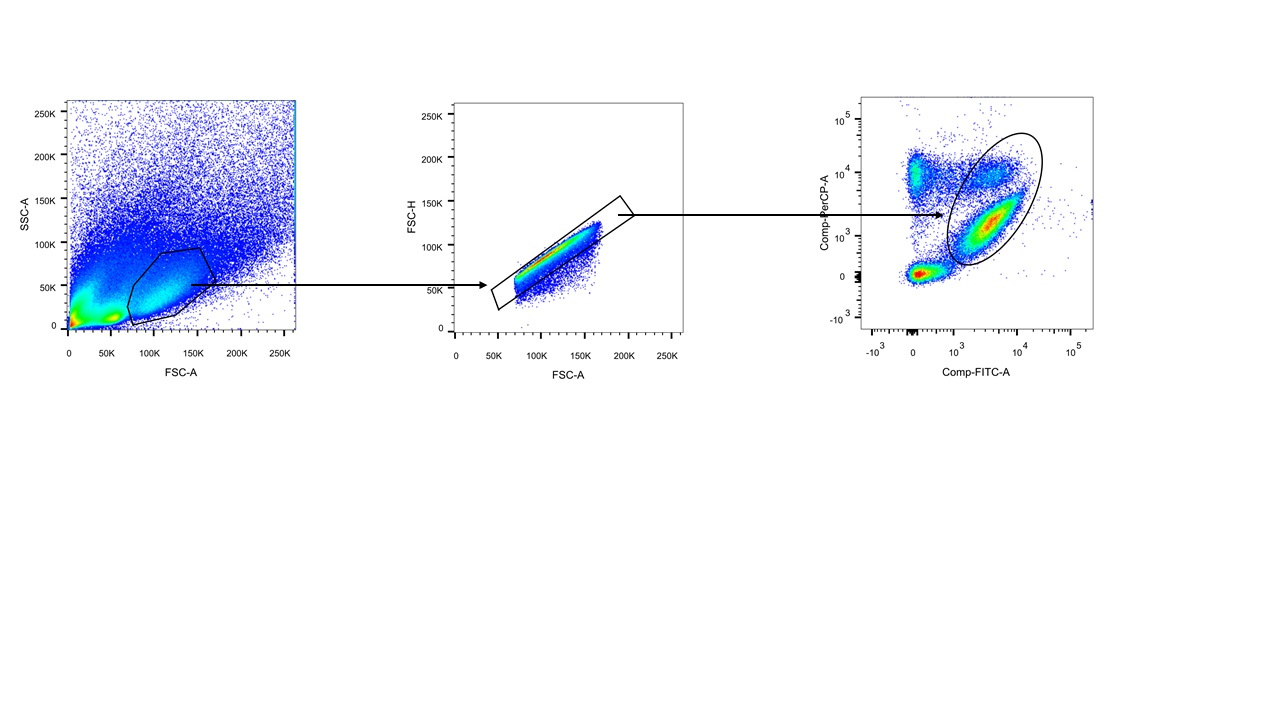

Supplement: Supplementary Figure S2 — FACS gating strategy.jpg. Gating strategy used to isolate microglia. [file Image_2.JPEG]

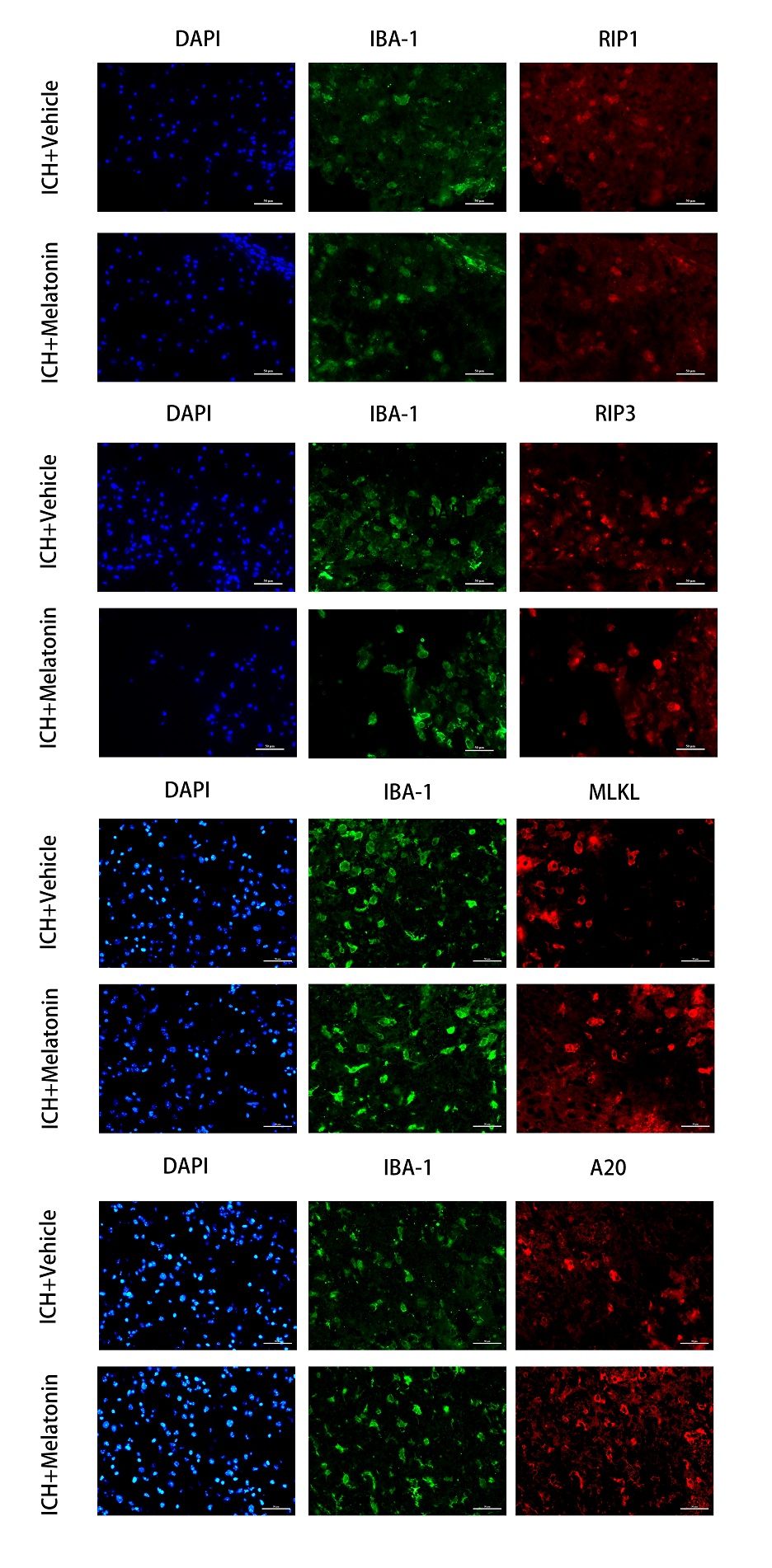

Supplement: Supplementary Figure S3 — Single-stained fluorescence.jpg. Single-stained high-magnification (40×) fluorescence images. [file Image_3.JPEG]
